# Material Characterization of Hardening Soft Sponge Featuring MR Fluid and Application of 6-DOF MR Haptic Master for Robot-Assisted Surgery

**DOI:** 10.3390/ma11081268

**Published:** 2018-07-24

**Authors:** Jong-Seok Oh, Jung Woo Sohn, Seung-Bok Choi

**Affiliations:** 1 Division of Mechanical & Automotive Engineering, Kongju National University, Cheonan-Si 31080, Korea; jongseok@kongju.ac.kr; 2 Department of Mechanical Design Engineering, Kumoh National Institute of Technology, Gumi-Si 39177, Korea; jwsohn@kumoh.ac.kr; 3 Smart Structures and Systems Laboratory, Department of Mechanical Engineering, Inha University, Incheon 22181, Korea

**Keywords:** hardening sponge, MR sponge, 6 degrees-of-freedom (6-DOF) MR haptic master, RMIS (robot-assisted minimally invasive surgery)

## Abstract

In this work, the material characterization of hardening magneto-rheological (MR) sponge is analyzed and a robot-assisted surgery system integrated with a 6-degrees-of-freedom (DOF) haptic master and slave root is constructed. As a first step, the viscoelastic property of MR sponge is experimentally analyzed. Based on the viscoelastic property and controllability, a MR sponge which can mimic the several reaction force characteristics of human-like organs is devised and manufactured. Secondly, a slave robot corresponding to the degree of the haptic master is manufactured and integrated with the master. In order to manipulate the robot motion by the master, the kinematic analysis of the master and slave robots is performed. Subsequently, a simple robot cutting surgery system which is manipulated by the haptic master and MR sponge is established. It is then demonstrated from this system that using both MR devices can provide more accurate cutting surgery than the case using the haptic master only.

## 1. Introduction

In recent years, haptic technology has led to advances in various fields of study including robotics, space exploration, manufacturing, and transportation. In particular, haptic technology has the potential to significantly influence medical industry such as robot-assisted minimally invasive surgery (RMIS) by providing surgeons with a sense of touch. Currently, a commonly used surgery robot system, such as da Vinci™, does not provide haptic sensation as feedback to a surgeon. So, the surgeon cannot perceive how strongly he or she is handling a patient’s organ. This may be quite dangerous for the patient. Hence, the restoration of reaction force feedback and tactile information is needed for safer and more accurate robotic surgery. In order to realize the haptic feedback, many types of haptic master and tactile device have been proposed [1,2,3,4,5,6,7,8,9,10,11,12,13,14,15,16,17,18,19,20,21]. However, RMIS systems with reaction force or tactile feedback showed limited performance. Thus, this study proposes a novel method using both force feedback and tactile sensation for application in RMIS.

Haptic master devices utilizing servomotors had been proposed to solve absence in haptic feedback [1]. However, many disadvantages of the proposed master are reported. For example, it is difficult to obtain continuous and smooth haptic feedback owing to the cogging phenomenon, brush friction, and so forth. Also, an active system using a servomotor is inherently unstable due to power failure and malfunction of the controller. By contrast, the configuration of a semi-active control system is similar with that of a passive system. So, the semi-active system shows passive performance when the power failure occurs. Accordingly, new approaches using controllable magneto-rheological (MR) fluids have been proposed to devise a controllable haptic master. It is generally known that the actuator using MR fluid can obtain smooth and continuous actuating motion owing to the phase change from the liquid to the solid state in a considerably short time by controlling a magnetic field. Several types of MR actuator have been commercially applied to obtain high performance, such as clutch, brake, and several types of damper [2,3,4,5,6,7,8]. Among them, the rotary damper and MR sponge damper is actively applied for vibration control application [7,8]. Using the salient property of the MR fluid, several types of haptic master systems have been proposed and investigated in multiple studies. Li et al. developed a haptic master using 2-degrees-of-freedom (DOF) MR brakes [9]. Senkal et al. developed a joystick-type 3-DOF haptic master featuring spherical brake mechanism [10]. Furthermore, a joystick with a 2-DOF haptic system was proposed by using an MR damper mechanism [11]. Oh et al. devised a haptic feedback system by integrating a 4-DOF haptic master using a bidirectional MR clutch and gimbal mechanism to enhance surgical accuracy [12,13]. In clinical practice, a higher-DOF haptic master system is requisite for accomplishing the motions required for surgery in any direction. In other words, the slave robot for RMIS is required to have 3 DOFs for human wrist motion (pitch, yaw, and roll) and 3 DOFs for robot manipulation motion (X, Y, and Z) to stretch any point or position of an incised organ. A few studies proposed a 6-DOF haptic master using servomotors for RMIS [14,15].

In addition, a few tactile devices for sensing the hardness of living tissue were reported for application to RMIS. Several studies on tactile devices that can be integrated with haptic masters have been performed for robot surgery [16,17]. However, these studies have also identified difficulty in realizing the various sensations of human organs by touching sensors. In order to resolve this limitation, in recent years, several types of tactile device with controllable MR fluid have been proposed. The viscoelastic property of the MR fluid can be controlled using the magnitude of the magnetic field; thus, a tactile device can generate various levels of stiffness (or softness) that can represent most human-like organs/tissues. Han et al. and Oh et al. developed a tactile device composed of a diaphragm and MR fluid and evaluated its effectiveness via a psychological test [18,19]. Even though an appropriate reaction force was generated, the viscoelastic features of organs could not be represented because it is not enough to express the elastic property of human tissues using MR fluid and a diaphragm. Given this, Kim et al. presented a hardening sponge device composed of MR fluid and an elastic sponge [20]. The desired reaction force and viscoelastic sensation could be easily realized by changing the current intensity of the MR sponge. The performance evaluation was undertaken using porcine specimens, whose viscoelastic features are similar to those of humans [21,22,23]. From test results, it was demonstrated that the MR sponge can mimic the tactile feeling of actual human-like organs through multiple experimental tests. Accordingly, it is expected that the MR sponge tactile device can help surgeons feel the stiffness of the organ/tissues during RMIS.

However, so far there has been no research in terms of a robot surgery experiment that utilizes both the 6-DOF MR haptic master and the MR tactile device simultaneously. Consequently, the technical novelty and contributions of this work are summarized as follows. As a first step, the inherent properties of MR fluid and sponge used in this work are characterized as a function of the magnetic field (or current) and deformation length to design an appropriate size of MR sponge device. Then, a cell-type of hardening sponge device is developed using a smart MR fluid and a sponge to generate various magnitudes of the force of human-like tissues. Its effectiveness is demonstrated via experimental tests performed on two specimens, i.e., the porcine heart, lung, and liver. Secondly, a new type of 6-DOF haptic master applicable in RMIS is developed using the following controllable fluid actuators: MR brakes for 3-DOF rotational motion and MR clutches for 3-DOF translational motion. This is the first work on the medical haptic master, which has 6 DOFs controlled by MR actuators. This geometry can decouple rotational motion from translational motion, making this a simple structure with decoupled dynamics. The proposed mechanism and design process of medical haptic systems can be applied to several types of high-DOF mechanisms. Lastly, to demonstrate the effectiveness of the haptic system, the cutting surgery operations of the porcine specimens are performed by eliminating a tumor section of the specimens, which is marked by dyeing it in black color.

## 2. Hardening Sponge Featuring Magneto-Rheological (MR) Fluid

**Figure 1:**
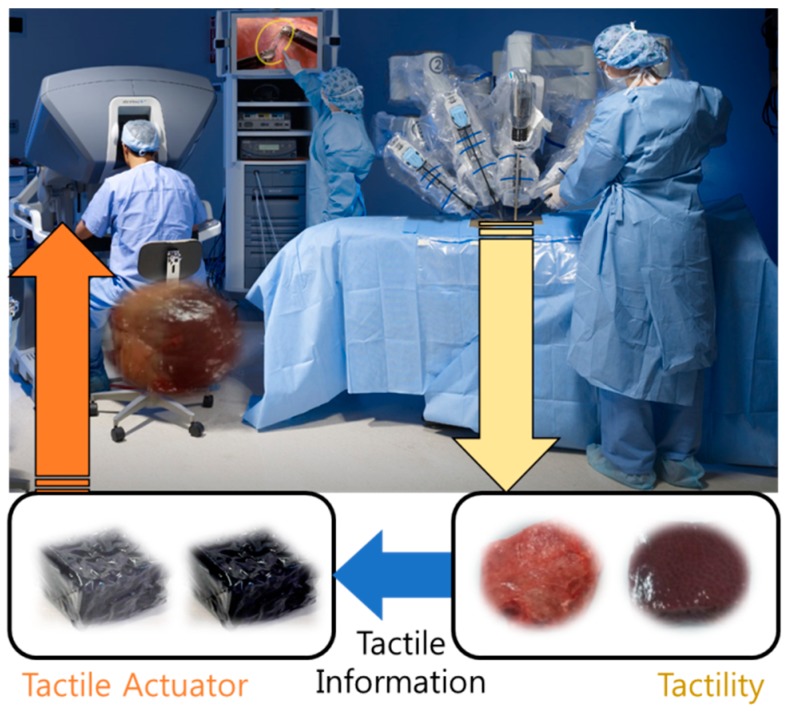
Concept of tactile sensation by the surgeon.

In this work, a novel tactile device featuring a hardening sponge is devised for RMIS. As a classical RMIS system cannot provide the viscoelastic properties of biological tissues or organs, the realization of tactile sensation can be helpful for surgeons, as shown in Figure 1. We introduce an MR fluid, sponge, and film into the proposed tactile device to mimic biological tissues or organs.

### 2.1. Force Measurement of Human-Like Tissues

**Figure 2:**
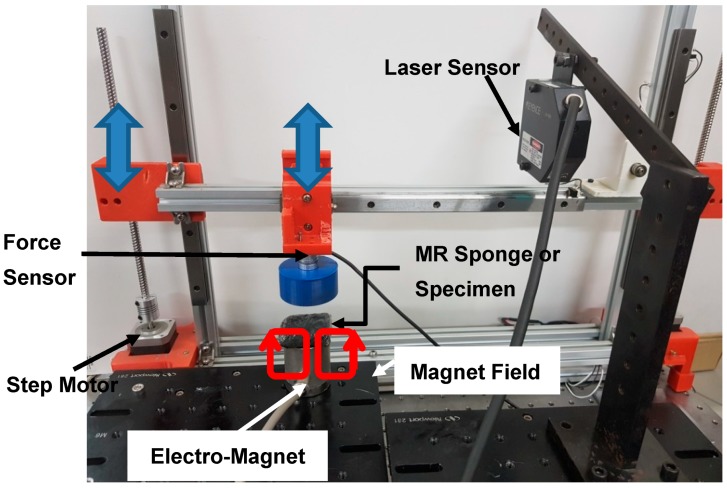
Photograph of force measurement system.

**Figure 3:**
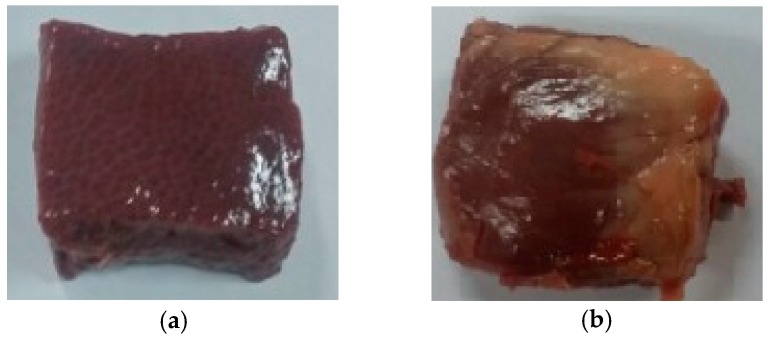
The specimens tested in this work; (**a**) porcine liver; (**b**) porcine heart.

When realizing the tactile sensation of organs or tissues, reaction force, texture, temperature, depth of impression, and weight can be considered. Among several indices, it is known that the reaction force between an organ and a human finger is an important factor for tactile sensation [18]. In this work, as the beginning research stage, only reaction force is selected as the tactile recognition index. As shown in Figure 2, a gantry-type robot with a force sensor is utilized to quantify the reaction force of organs and tissues. When the surgeon touches the organ, the operation speed is very slow. So, that process is considered to be the quasi-static process and the travel speed of end-effector is restricted to 0.4 mm/s in this study. For more information about the gantry robot, please refer to our previous study [24]. Two lead screws with step motors are placed in the vertical direction. A guide rail is horizontally placed between the lead screws. A timing belt, a step motor, and an end effector are mounted on the guide rail. Owing to this mechanism, the position of the end effector moves along the *z*-axis and *x*-axis directions. When the end effector compresses the specimen, palpation force is measured by a force sensor (ATI Corp., Nano17, Apex, NC, USA). The resolution of the force sensor is 0.0125 N. The maximum moving speed of the end effector is maintained low (0.4 mm/s) to consider only the quasi-static process. After the specimen is deformed up to 1 mm, the end effector is stopped and the force history is measured during 30 s. This is because a surgeon requires a specific time to feel unknown objects. As shown in Figure 3, the porcine heart and liver are selected as specimens. It should be noted that the features of porcine organs are largely similar to those of human tissues. The dimensions of the specimens are 25 × 25 × 10 mm^3^ (width × height × thickness). Generally, the stiffness and damping of deformable objects are mainly determined by the reaction force along the normal direction [20]. Thus, the end effector with the force sensor touches the specimen perpendicularly.

**Figure 4:**
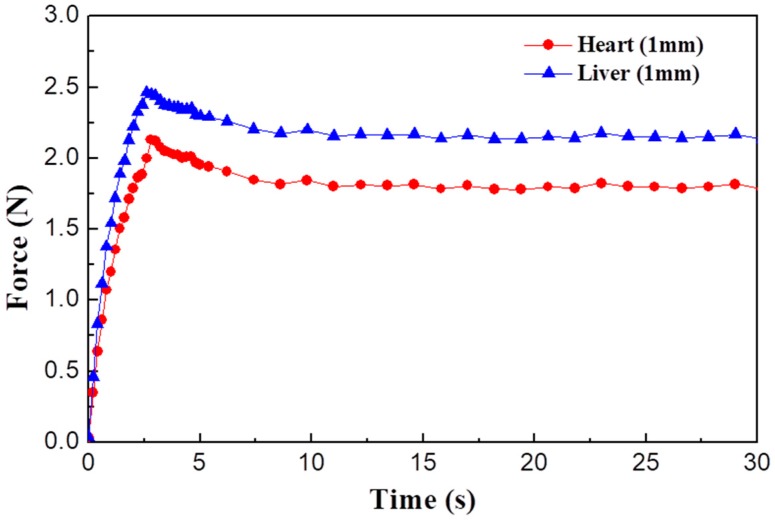
Force levels of two specimens.

Figure 4 shows the measured results of the two specimens. Reaction force increases until 2.5 s and then decreases exponentially. As mentioned earlier, the displacement of the end effector is 1 mm and travel time is 2.5 s. If the specimen is created from a purely elastic material, reaction force is proportional to deformation length. Thus, the behavior of a purely elastic material cannot represent the reduction in reaction force. In addition, the reaction force of a purely viscous material is almost zero for constant deformation. Accordingly, it is considered that the physical features of the specimens are similar to that of a viscoelastic material. It is known that viscoelastic materials show viscous and elastic characteristics [25]. It is observed from Figure 4 that the stiffness of the porcine heart is the largest based on the magnitude of reaction force. Additionally, the rate of reduction in force and the gap between the maximum and equilibrium values are different for each specimen.

### 2.2. Material Property of Sponge and MR Fluid

In order to mimic the tactile feeling of an organ, a material that has similar viscoelastic material properties should be used. Sponge is generally known as a viscoelastic material but the material property is constant. Since human organs and tissues have diverse reaction force characteristics as mentioned above, several sponge materials are required for surgery. During RMIS, it is very difficult to change the sponge according to the organ or tissue. Accordingly, a hardening sponge device featuring MR fluid is devised to realize several force characteristics. The phase of the MR fluid which consists of micron-sized iron particles and carrier fluid can be changed under a magnetic field. This phenomenon is due to the polarization generated in the iron particles. Owing to the chain structures, the reaction force of the MR sponge is tuned according to the intensity of the magnetic field.

**Figure 5:**
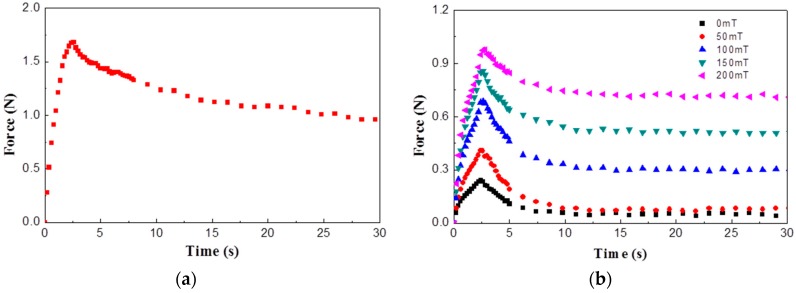
Measured reaction force results; (**a**) polyurethane foam; (**b**) magneto-rheological (MR) fluid.

Open-celled polyurethane (PU) foam is selected as the material of the sponge. Foam is a cellular structure and open-celled foams consist of numerous pores in an interconnected network. The foam has 25 pores per inch (ppi) and the reaction force of the polyurethane foam is measured with the same test conditions and gantry robot system. Figure 5a shows the measured force of the sponge only. The maximum force is 1.68 N which is smaller than that of heart and its force characteristic shows the viscoelastic behavior. From the test results, it is known that the open-celled PU foam can be classified as a soft viscoelastic material.

MRF-132DG fluid provided by Lord Corp. (Cary, NC, USA) is used in this study and its reaction force during squeeze mode is measured. Figure 5b shows the measured force with several magnetic field inputs and deformations. Since the remaining force value is almost zero without magnetic field input, the behavior of MR fluid is similar to that of viscous material. However, the behavior of MR fluid is changed to that of viscoelastic material according to the magnetic field input. It is generally known that MR fluid behaves like a Newtonian fluid without a magnetic field. If a magnetic field is applied to an MR fluid, the iron particles are aligned with the direction of the magnetic field and form chain structures. It is inferred from test results that the chain structure of MR fluid is related to the measured elastic force characteristics.

### 2.3. Fabrication of MR Sponge

**Figure 6:**
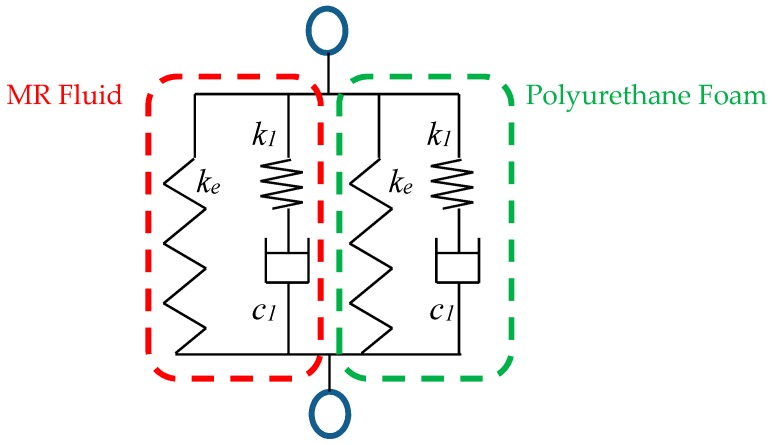
Mechanical model of MR sponge device.

In order to take advantages of MR fluid and polyurethane foam, an MR sponge device is devised. Since, the MR fluid sinks into the polyurethane foam, the mechanical configuration of the MR sponge can be illustrated by spring and dashpot elements as shown in Figure 6. The dashpot and spring mean the viscous and elastic components of the material, respectively. *k* is the spring coefficient of the each materials and *c* is its damping coefficient. The MR fluid and polyurethane foam are connected in parallel. From the mechanical model, the following equations are derived:

(1) $$\begin{array}{l} F_{1}=k_{1}x_{1}=c_{2}{\dot{x}}_{2}=F_{2}\\ F_{e}=k_{e}x_{total}\\ x_{total}=x_{1}+x_{2}\\ F_{total}={\left(F_{e}+F_{1}\right)}_{MR}+{\left(F_{e}+F_{1}\right)}_{sponge} \end{array}$$

where *x*_1_, *x*_2_ are deformed displacements of spring and damping elements in series connection. *F*_1_ and *F*_2_ are spring and damping forces in series connection. The magnitudes of the two forces are the same due to the law of action and reaction. From Equation (1), it can be inferred that the total reaction force of the MR sponge device is expected to be the sum of reaction forces of MR fluid and polyurethane foam. By using the proposed force model, the MR sponge device can be designed to meet the required force magnitudes. It is noted that the spring and damping constants in Equation (1) are not obtained in this work. From the measured reaction force results in Figure 4, the required peak force range is 2–2.5 N.

**Figure 7:**
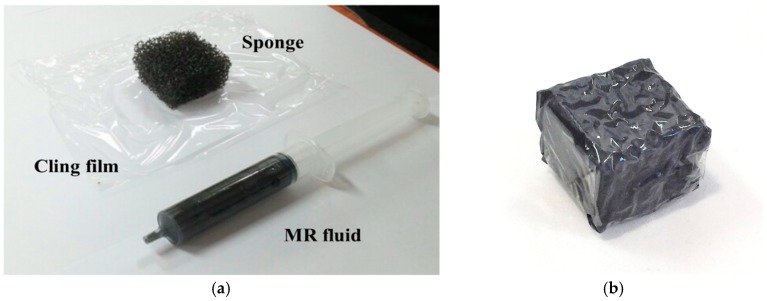
MR sponge tactile device; (**a**) components; (**b**) assembly.

The size of the polyurethane foam is 25 × 25 × 10 mm^3^. A slippery film clings to the sponge to prevent leakage of the MR fluid. Figure 7 shows the components and assembly of the single MR sponge cell. After the MR sponge is deformed, its original shape is recovered owing to its elasticity. During deflection and restoration, the repulsive force between the MR sponge and a surgeon is controlled by a magnetic field. Based on this mechanism of the MR sponge, the surgeon can distinguish between the tactile sensations of various cells and biological organs. Because the surgeon remotely manipulates the slave robot via the haptic master, the mechanism of the tactile device is extremely helpful to the surgeon in accomplishing accurate surgery.

**Figure 8:**
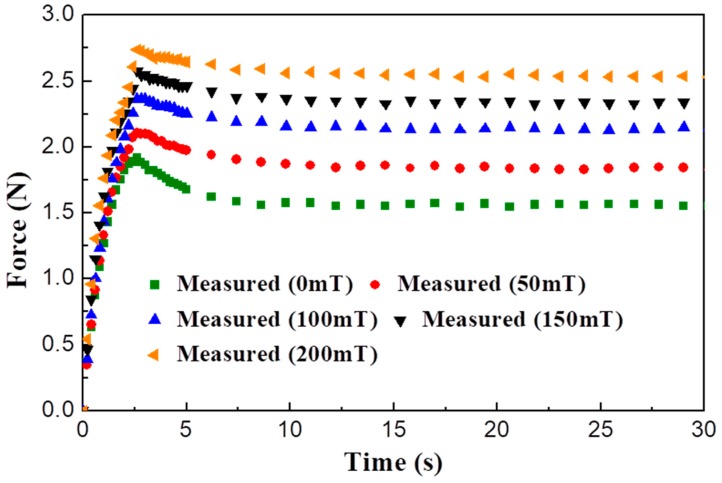
The field-dependent force of MR sponge.

The reaction force measurement test of the MR sponge is conducted to mimic these features of the specimens. An electromagnet (JL-4A, JL Magnet Corp., Seoul, Korea) is used to apply magnetic fields of several intensities to the MR sponge. The reaction force of the MR sponge can be tuned because the MR fluid is affected by the magnetic field. As shown in Figure 8, the maximum reaction force increases according to the magnetic field. It can be inferred that the MR sponge is a viscoelastic material and the reaction force curves can be controlled by magnetic inputs. Hence, the devised MR tactile device can realize a wide range of the tactile sensations of organs or tissues. For instance, when a magnetic field of 50 mT is applied to the MR sponge, then the reaction force curve is almost similar to that of the porcine liver. Also, the predicted maximum forces according to magnetic field inputs are 1.91 N, 2.08 N, 2.35 N, 2.52 N, and 2.65 N, respectively. The mean error percentage between measured and predicted maximum forces is 1.15%. These results show the effectiveness of the proposed force model. But this model is only valid for transient response such as peak force. In order to predict the force characteristics in a steady state response, the time constant and compression region should be considered. For more detailed information, please refer to our previous study [24].

## 3. 6 Degrees-of-Freedom (DOF) Haptic Master with MR Actuators

**Figure 9:**
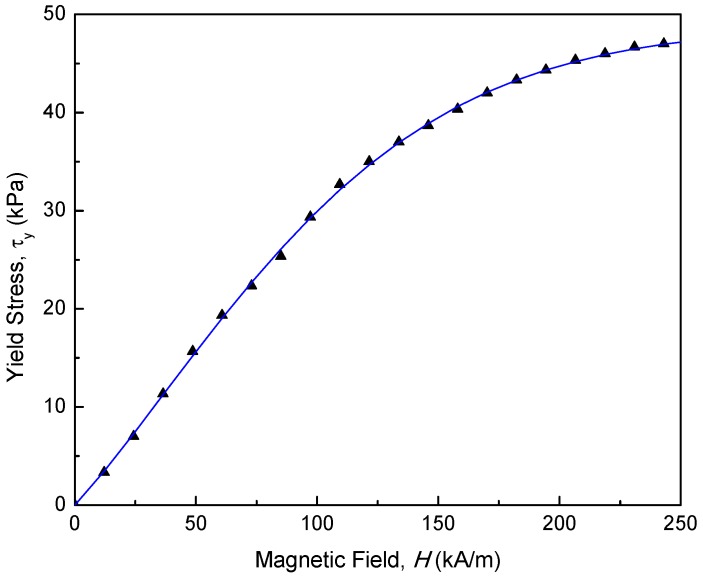
MRF-132 DG τ-*H* curve.

The shear stress of MRF-132DG can be expressed based on the Bingham model:

(2) $$\tau =\eta \dot{\gamma }+\tau_{y}\left(B\right)$$

where η and $\dot{\gamma }$ are viscosity constant and shear rate, respectively. The nominal value of the viscosity constant is 0.092. $\tau_{y}\left(B\right)$ is yield stress of MR fluid which is changed according to the magnitude of the magnetic field, *B*. Lord Corp. presents the yield stress properties of MRF-132DG [26]. When MRF-132DG is shearing between two surfaces, the shear stress is generated and measured. The MR fluid’s particles align with the direction of the magnetic field, thereby restricting the fluid’s rotational motion within the gap in proportion to the strength of the magnetic field. From Figure 9, the relation between yield stress and magnetic field is obtained by using the curve-fitting method.

(3) $$\begin{array}{l} \tau_{y}\left(B\right)=m_{MR0}B^{5}+m_{MR1}B^{4}+m_{MR2}B^{3}+m_{MR3}B^{2}+m_{MR4}B^{1}+m_{MR5}\\ where\\ m_{MRi}=\left[\begin{array}{ccccc} -4.1319\times 10^{-11} & 4.1437\times 10^{-08} & -1.39\times 10^{-05} & 1.1\times 10^{-03} & \begin{array}{cc} 2.886\times 10^{-01} & -1.178\times 10^{-01} \end{array} \end{array}\right] \end{array}$$

It is noted that the above equation for the field-dependent property of MR fluid is to be used to determine appropriate dimensions of the haptic master.

**Figure 10:**
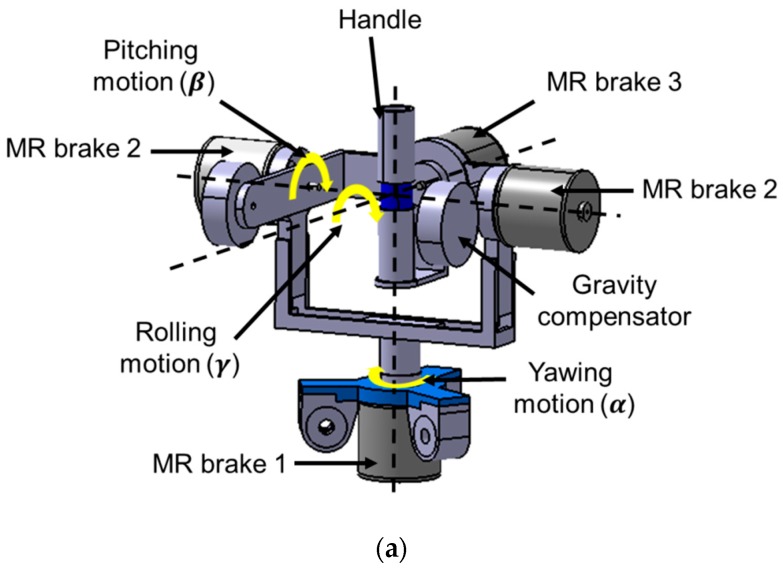

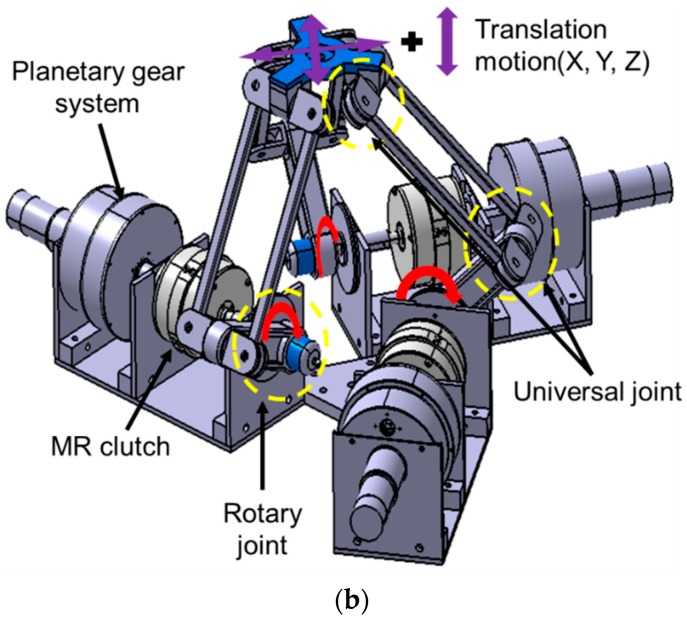
Overall configurations of the haptic master; (**a**) rotational part; (**b**) translational part.

**Figure 11:**
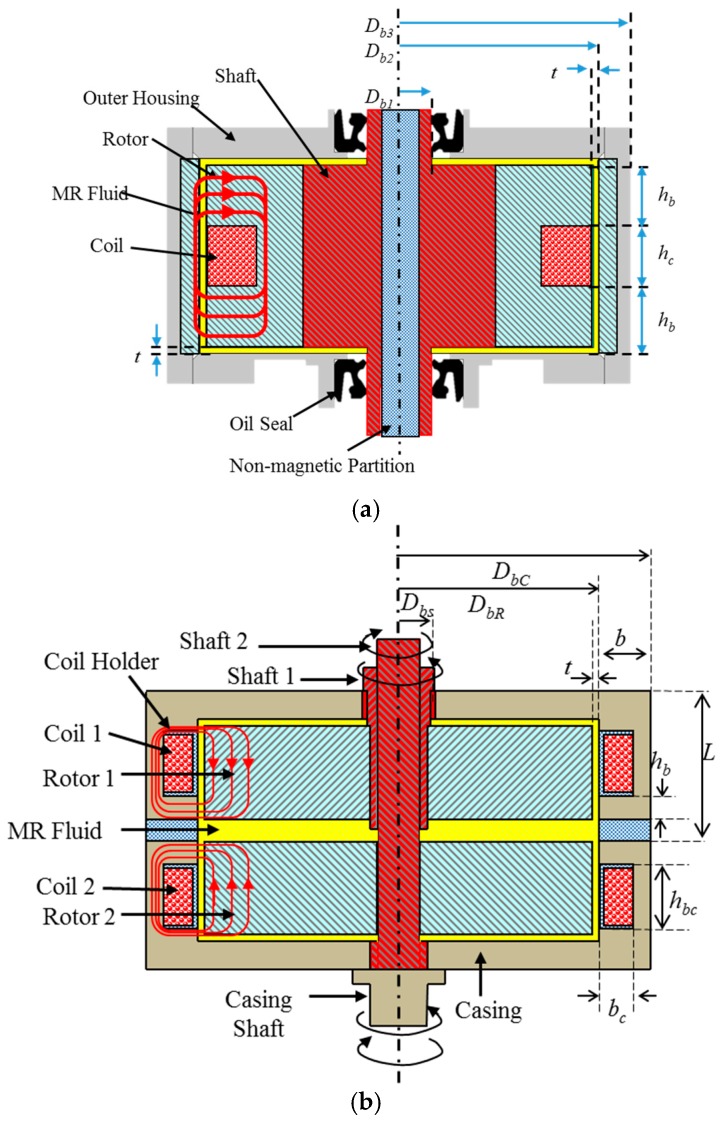
Internal configuration of MR devices; (**a**) MR brake; (**b**) MR clutch.

It is generally known that RMIS requires 6-DOF motions (3-DOF surgical instrument motions and 3-DOF end effector motions) [14,15]. A 6-DOF MR haptic master is proposed to realize 6-DOF surgical motions and reaction force/torque, as shown in Figure 10. When a surgeon grips and manipulates the handle, the handle’s 6-DOF motion command is transferred to the surgical robot, and the reaction force between the surgical instrument and organ/tissue should be provided by the haptic master. Thus, the MR actuator (clutch/brake) for each motion is required to supply reaction force to the surgeon, and several MR actuators are integrated with the motion mechanism of the haptic master. Based on the parallel robot mechanism, 3-DOF translational motions are realized using a moving platform, 6 links, and rotary/universal joints [27]. The handle and moving platform are connected to each other. Hence, when the handle moves along the X, Y, and Z axes, the moving platform and links are moved but the MR clutch rotates without moving. It is noted that the torque induced from the MR clutch is transferred to the surgeon as reaction force via the handle. In addition, 3-DOF rotational motions (pitching, rolling, and yawing) and reaction torque are realized at the handle. When the handle is rotated, the MR brake generates reaction torque against rotational motion. An additional counter mass is attached to the other side of the MR brake. The purpose of the counter mass is to prevent the handle from rotating owing to its mass. Figure 11 shows the configuration of the MR brake, which mainly consists of an inner rotor, an MR fluid, a coil, and an outer casing. When the inner rotor rotates, fluid friction, which is determined by the shear stress of the MR fluid, is induced. Because this fluid friction is converted to reaction torque, the shear stress of the MR fluid can determine the reaction torque generated by the MR brake.

The reaction torque of the MR brake can be expressed as follows:

(4) $$\begin{array}{l} T_{b}=T_{c}+T_{\eta }+T_{f}\\ =\left[2\frac{\pi D_{b2}{}^{2}h_{b}\tau_{y}\left(B\right)}{2}+\frac{\pi \left(D_{b2}{}^{3}-D_{b1}{}^{3}\right)\tau_{y}\left(B\right)}{12}\right]\\ +\left[2\frac{\pi \eta D_{b2}{}^{3}h_{b}\left|\dot{\gamma }\right|}{4t_{b}}+\frac{\pi \eta \left[{\left(\frac{D_{b2}}{2}\right)}^{4}-{\left(\frac{D_{b1}}{2}\right)}^{4}\right]\left|\dot{\gamma }\right|}{2t_{b}}\right]+T_{f} \end{array}$$

where, *T_c_* is the controllable torque induced from yield stress, *T_η_* is the fluid friction torque induced from viscosity of the MR fluid. *T_f_* is mechanical friction torque induced from oil seal. Contrary to the MR brake mechanism, the MR clutch mechanism requires a driving actuator to generate reaction torque. Thus, two shafts and two rotors are connected to each other. When one shaft rotates, one rotor rotates and the torque of the one rotor is transferred to the outer housing via the MR fluid. Thus, if the rotational directions of the shafts are different, then the rotational direction of the rotor is different. In addition, the rotational direction of the outer housing is determined by the difference between the torques transmitted from each rotor. Similar to the mechanism of the MR brake, the magnitude of transmitted torque is determined by the shear stress of the MR fluid. Based on the configuration of the MR clutch, reaction torque can be expressed as follows:

(5) $$T_{total}=\left|{\vec{T}}_{1}-{\vec{T}}_{2}\right|,\;T_{i}=T_{ci}+T_{\eta i}+T_{fi},\;i=1,2$$

where *T*_1_ and *T*_2_ are the generated torque between each rotor and outer housing. From Equation (5) and configuration of the MR clutch, the total torque model is derived as follows:

(6) $$T_{i}=2\pi {\left(\frac{D_{bR}}{2}\right)}^{2}\int_{0}^{h_{b}}\tau \;dz+2\pi \int_{\frac{D_{bs2}}{2}}^{\frac{D_{bR}}{2}}r^{2}\tau dr+T_{f}$$

**Figure 12:**
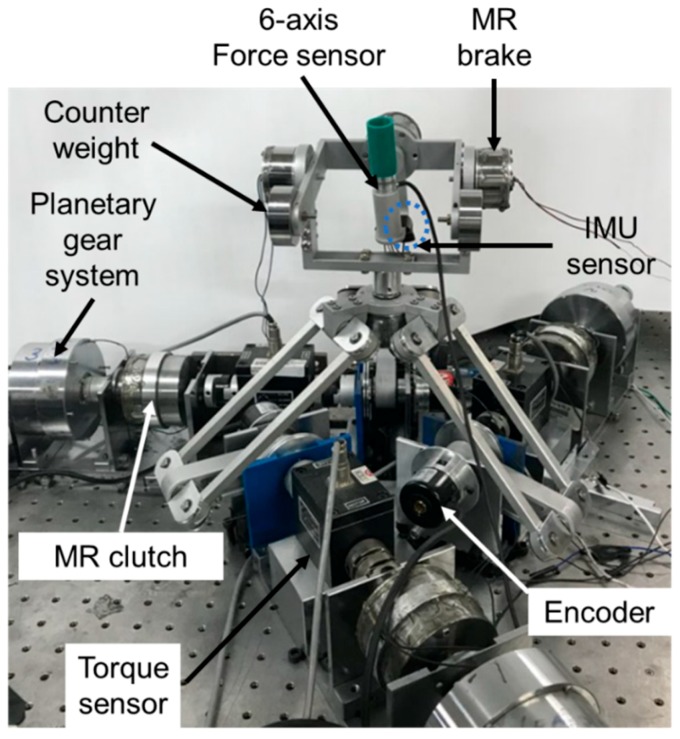
Photograph of the haptic master system featuring MR clutches and brakes.

It is noted here that all variables are denoted in Figure 11. On the other hand, a DC motor and a planetary gear system are employed to supply driving power to the MR clutch. When the DC motor rotates along one direction, rotation in the opposite direction is generated via the planetary gear mechanism. The two output shafts are connected with each shaft of the MR clutch. If the MR brake is replaced with the MR clutch and planetary gear, then the weight becomes extremely high. When the reaction force along the upper direction is necessary, a higher mass of the handle requires a large MR clutch. The MR brake mechanism is applied at the handle to obtain a compact size of the MR haptic master system. Based on multiple studies [28,29], the maximum reaction torque and force were set at 0.5 Nm and 12 N, respectively. The reaction torque of the MR clutch is transformed to the reaction force along the translation motions at the handle. Hence, the objective torques of the MR brake and clutch are set to be 0.75 and 2.5 Nm, respectively. The design parameters calculated based on the generated torque model are listed in Table 1. Please refer to our previous study [19,30] for more details about the process. The diameters of the outer housing for the MR clutch and brake are 50 mm and 34 mm, respectively. Finally, the MR haptic master is manufactured as shown in Figure 12. In addition, an encoder (E40H; Autonics, Incheon, Korea) and an IMU sensor (MPU-9250, InvenSense, Sunnyvale, CA, USA) are used to measure position information; a 6-axis force/torque sensor and a 1-DOF torque sensor (SDS-100; Senstech Corp, Busan, Korea) are used for torque and force measurement.

## 4. Robot Surgery Experiment

### 4.1. Integration of Haptic Master and Slave Robot

**Figure 13:**
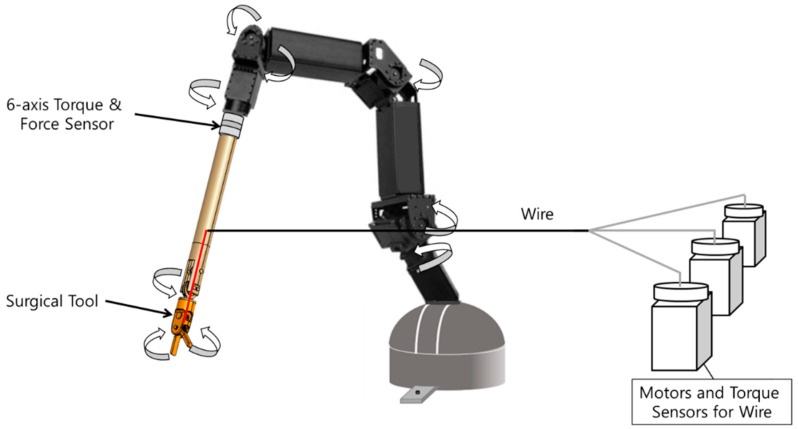
Overall configuration of the slave robot.

As mentioned earlier, the slave robot implements the surgical command transferred from the master. The mechanism of the 6-DOF slave robot is proposed as shown in Figure 13. The proposed slave robot consists of three robot arms, servomotors, and a surgical instrument. Because the surgical instrument is inserted into a small incision, the instrument is attached to the end point of the third robot arm. In addition, a surgical tool such as forceps or a knife is placed at the opposite end of the instrument. As the wire and motor actuator are connected with the surgical tool, the surgical tool can rotate in three directions, i.e., rolling, pitching, and spinning. Each robot arm can rotate using the servomotor, and the end position of the third arm moves along the X, Y, and Z axes. The position of the end point is determined according to the translation motion of the moving platform of the haptic master. Based on the forward kinematics of the 3-DOF manipulator, the position of the moving platform, $P_{mx},P_{my},P_{mz}$, can be expressed as follows [27]:

(7) $$\begin{array}{l} P_{mx}=\frac{1}{h_{22}h_{33}-h_{23}h_{32}}\left((h_{32}h_{43}-h_{33}h_{42})P_{mz}+h_{23}h_{42}-h_{22}h_{43}\right)\\ P_{my}=\frac{1}{h_{22}h_{33}-h_{23}h_{32}}\left((h_{13}h_{32}-h_{12}h_{33})P_{mz}+h_{12}h_{23}-h_{13}h_{22}\right)\\ P_{mz}=l_{1}\sin\theta_{11}+l_{4}\sin\theta_{21}=l_{2}\sin\theta_{12}+l_{5}\sin\theta_{22}=l_{3}\sin\theta_{13}+l_{6}\sin\theta_{23}\\ h_{1j}=-2\cos\phi_{j}(l_{1}\cos\theta_{1j}+a-b)+2\cos\phi_{j}(l_{2}\cos\theta_{2j}+a-b)\\ h_{2j}=-2\cos\phi_{j}(l_{1}\cos\theta_{1j}+a-b)+2\sin\phi_{j}(l_{2}\cos\theta_{2j}+a-b)\\ h_{3j}=-2l_{1}\sin\theta_{1j}+2l_{2}\sin\theta_{2j}\\ h_{4j}={\left(l_{1}\cos\theta_{1j}+a-b\right)}^{2}+l_{1}{}^{2}\sin^{2}\theta_{1j}-{\left(l_{2}\cos\theta_{2j}+a-b\right)}^{2}-l_{2}{}^{2}\sin^{2}\theta_{2j}\\ where\;j=1,2,3 \end{array}$$

In Equation (7), *l* is the length of the 6 lower legs attached to the moving platform. $\phi_{j}$ is the angle of each lower leg on the XY plane. $\theta_{1j},\theta_{2j}$ are the angles between each lower leg. *a* is the displacement between the center and each lower leg, and *b* is the radius of the moving platform. When the position of the moving platform is measured by the encoder, the rotation angle of the servomotor for the slave robot is obtained based on inverse kinematics. In addition, the rotation information of the haptic master handle along the pitching, rolling, and spinning directions is directly transferred to the surgical tool part of the slave robot. Based on the transferred rotation command, the surgical tool is rotated using the wire actuator. Please refer to the details about the transformation in [31,32]. The rotational information of the handle is measured using the inertial measurement unit (MPU-9250).

### 4.2. Experimental Results and Discussion

**Figure 14:**
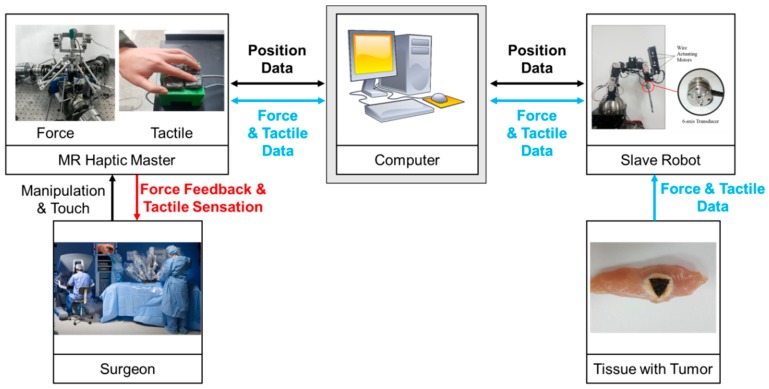
Experimental configuration for the robot surgery integrated with MR haptic master and tactile device.

A tumor cutting experiment was performed to evaluate the performance of the proposed system. As it is extremely important to clearly distinguish between a tumor and normal tissue, an efficient method, such as haptic feedback and tactile sensation, is required. Two testing conditions are adopted to maximize the performance of the MR tactile device. In the first condition, participants cut tumors using only the haptic feedback from the MR haptic master, whereas in the second condition, they use the haptic feedback and tactile sensation from the MR tactile device. Figure 14 shows the experimental apparatus of the overall cutting surgery system, which is integrated with the MR haptic master, MR tactile device, and slave robot. When a participant manipulates the MR haptic master, translational and rotational commands are transferred to the slave robot via data acquisition boards (PXIe-6363, NI Corp., Austin, TX, USA) and A/D and D/A boards (PXIe-1082, NI Corp., Austin, TX, USA). The torques induced from the MR clutch are measured by the torque sensors (SDS-100). While implementing surgical tasks according to the commands, the slave robot transmits the reaction force between the specimen and surgical tool. This force is measured by the force sensor (Nano 17). The movement of the haptic master is measured by the encoders (E40H) and an IMU. Then, the desired angle for the slave robot is obtained based on Equation (4) and inverse kinematics. The servo motor of the slave robot rotates according to the calculated command and measured actual rotation angle. In order to reduce the position tracking error, a simple proportional-integral-derivative (PID) controller is implemented.

Prior to cutting surgery, the tracking control of the end-point of the slave robot by the haptic master is evaluated. The maximum error during the experiment is 4.02 mm. Based on the error results, the position tracking controller shows excellent performance. While implementing the surgical tasks according to the commands, the slave robot transmits the reaction force between specimen and surgical tool, which is measured by the force sensor. This force sensor is attached at the end point of the slave robot arm. The generated torque/force induced from the MR haptic master are measured by torque sensors and 6-axis torque/force sensor. In order to realize the measured force of the specimen, the torque/force tracking controller featuring the PID controller is proposed. Also, the input magnetic field for the MR tactile device is determined based on the relation between the reaction force and magnetic field.

**Figure 15:**
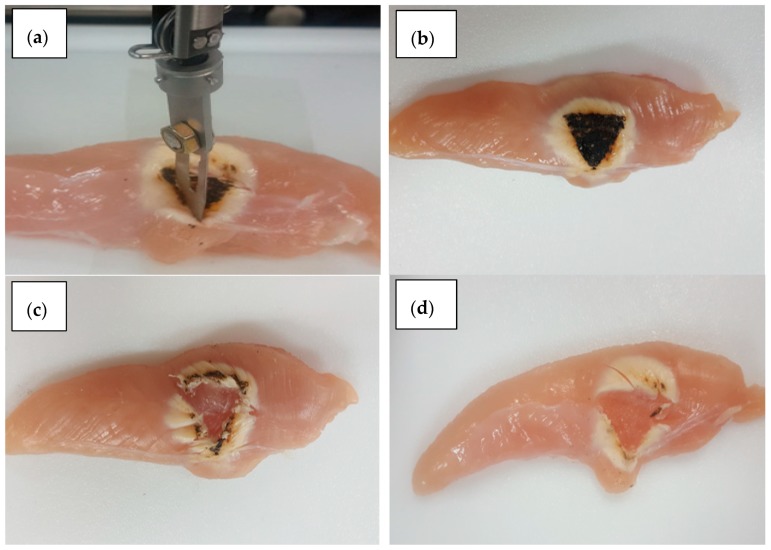
Surgical results; (**a**) cut operation; (**b**) tumor mark; (**c**) cut result with the haptic force only; (**d**) cut result with the haptic force and tactile sensation.

**Figure 16:**
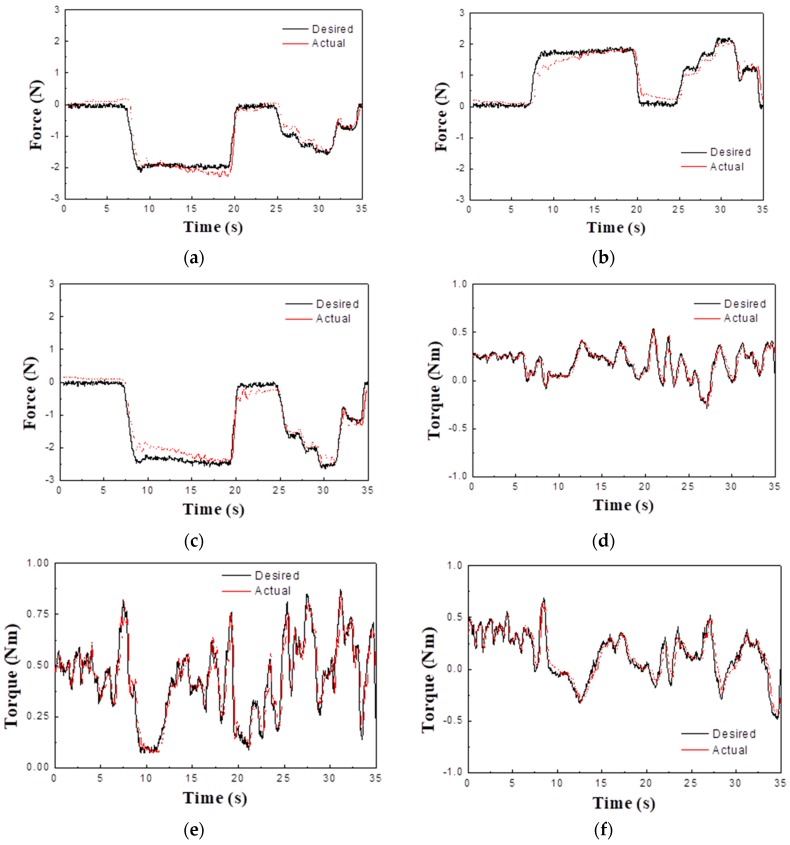
Force/torque tracking control results of the haptic master; (**a**) *x*-axis force; (**b**) *y*-axis force, (**c**) *z*-axis force; (**d**) pitching torque; (**e**) rolling torque; (**f**) spinning torque.

In order to undertake simple cutting surgery, a tumor is marked by dyed black in the specimen, as shown in Figure 15. The objective of the cutting experiment is to remove the black tumor from the normal tissue. It is generally known that the stiffness of the tumor and normal tissue are different. In order to emulate this, the forces of the porcine liver and heart tissues are used to replace the tumor and normal tissues in this experiment, respectively. When a magnetic field of 50 mT and 100 mT are applied to the MR sponge, then the reaction force curve is almost similar to that of the porcine liver and heart. During the cutting experiment, the haptic master generates the reaction torque/force from the MR brake/clutch by applying a magnetic field as input. At the same time, the position tracking controller of the slave robot is implemented, followed by a certain surgical operation. The operator feels the reaction force through the solidification of the MR fluid by the MR haptic master. In addition, the MR tactile device mimics the stiffness of the surgery specimen using the MR fluid effect. So, the operator can distinguish the tumor and normal tissues precisely. It can be clearly seen from Figure 15 that the use of the tactile device can increase surgical accuracy since the operator (surgeon) can feel the same stiffness of the surgical tissues from the proposed MR sponge tactile device. During cutting surgery with the haptic feedback force and the tactile sensation, the torque/force tracking control results of the MR actuators are measured and plotted, as shown in Figure 16. From the control results along three translation directions and 3 rotational directions, it can be clearly observed that the accuracy of the proposed control haptic master system is acceptable. Note that 20 specimens are used for the removal of the tumor in this surgical cutting experiment.

Since the usefulness of the tactile device cannot be measured simply, a psychological test is implemented to evaluate the effectiveness of the proposed tactile device for cutting surgery. Before cutting the tumor, the stiffness of tissues is measured via the force sensor of the slave robot. Then, the stiffness of the touched tissue is realized by the proposed tactile device and the participants are requested to touch the MR sponge and rate the following question on a five point scale.

- Question: Can you distinguish the different stiffness represented by the tactile device?
- Answer: (Negative) 1 ------- 2 ------- 3 (Mean) ------- 4 ------- 5 (Positive)

The number of participants was 30 and the age of participants ranged from 20 to 30. The mean value of answers was 4.5 and the standard deviation was 0.56. This result indicates that the proposed tactile device is very helpful in distinguishing the different stiffness of human organ/tissue for robot-assisted surgery.

## 5. Conclusions

In this work, novel types of smart devices are devised based on the control ability of MR fluid. As for the haptic master, MR clutches and MR brakes are used as actuators for the three translational motions and the three rotational motions, respectively. On the other hand, as for the tactile device, an MR sponge which can emulate the similar stiffness and/or damping property of human organs is devised. The overall network for the robotic surgery has been established by integrating the haptic master, MR sponge tactile device, slave robot and microprocessor including the PID control algorithm. Then, in order to achieve interactive motion between the command from the master followed by the slave robot, kinematic analysis was performed. As a final step, a tumor removal experiment using the porcine specimens was performed to demonstrate the superior performance of the proposed robot-assisted surgical system. It has been demonstrated via the experiment that the addition of the tactile device from which the operator (surgeon) can feel the same stiffness of the surgical object can enhance surgical accuracy. This has also been confirmed by a psychological test. Finally, it can be remarked that proposed haptic master and tactile device showed superior control performance due to the MR fluid and also the other control device can be designed based on the control ability of the MR fluid.

## Figures and Tables

**Table 1:** Design parameters of the haptic master actuators.

MR Clutch			MR Brake		
Parameter	Explanation	Value	Parameter	Explanation	Value
$D_{bR}$	Diameter of the MR clutch rotor	50 mm	$D_{b1}$	Internal diameter of the MR brake rotors	4 mm
$D_{bc}$	Diameter of the MR clutch’s outer housing	61 mm	$D_{b2}$	External diameter of the MR brake rotors	34 mm
$D_{bs}$	Diameter of the MR clutch’s shaft	10 mm	$h_{b}$	Height of the rotor	7 mm
t	Gap of the MR fluid	1 mm	$h_{c}$	Height of the coil	10 mm

## References

[1] Pierrot F., Dombre E., Dégoulange E., Urbain L., Caron P., Boudet S., Mégnien J.L. (1999). Hippocrate: A safe robot arm for medical applications with force feedback. Med. Image Anal..

[2] Nguyen Q.H., Choi S.B. (2008). Optimal design of a vehicle magnetorheological damper considering the damping force and dynamic range. Smart Mater. Struct..

[3] Nguyen Q.H., Choi S.B. (2010). Optimal design of an automotive magnetorheological brake considering geometric dimensions and zero-field friction heat. Smart Mater. Struct..

[4] Shafer A.S., Kermani M.R. Design and validation of a magneto-rheological clutch for practical control applications in human-friendly manipulation. Proceedings of the 2011 IEEE International Conference on Robotics and Automation (ICRA).

[5] Senkal D., Gurocak H. Compact MR-brake with serpentine flux path for haptics applications. Proceedings of the World Haptics 2009—Third Joint EuroHaptics Conference and Symposium on Haptic Interfaces for Virtual Environment and Teleoperator Systems.

[6] Nguyen P.B., Choi S.B. (2011). A new approach to magnetic circuit analysis and its application to the optimal design of a bi-directional magnetorheological brake. Smart Mater. Struct..

[7] Tusset A.M., Janzen F.C., Piccirillo V., Rocha R.T., Balthazar J.M., Litak G. (2018). On nonlinear dynamics of a parametrically excited pendulum using both active control and passive rotational (MR) damper. J. Vib. Control.

[8] Ulasyar A., Lazoglu I. (2018). Design and analysis of a new magneto rheological damper for washing machine. J. Mech. Sci. Technol..

[9] Li W.H., Liu B., Kosasih P.B., Zhang X.Z. (2007). A 2-DOF MR actuator joystick for virtual reality applications. Sens. Actuators A Phys..

[10] Senkal D., Gurocak H. (2009). Spherical brake with MR fluid as multi degree of freedom actuator for haptics. J. Intell. Mater. Syst. Struct..

[11] Ahmadkhanlou F., Washington G.N., Bechtel S.E. (2009). Modeling and control of single and two degree of freedom magnetorheological fluid-based haptic systems for telerobotic surgery. J. Intell. Mater. Syst. Struct..

[12] Song B.K., Oh J.S., Choi S.B. (2014). Design of a new 4-DOF haptic master featuring magnetorheological fluid. Adv. Mech. Eng..

[13] Oh J.S., Choi S.H., Choi S.B. (2014). Design of a 4-DOF MR haptic master for application to robot surgery: Virtual environment work. Smart Mater. Struct..

[14] Qiu T., Hamel W.R., Lee D. Design and control of a low cost 6 DOF master controller. Proceedings of the 2014 IEEE International Conference on Robotics and Automation (ICRA).

[15] Chen X., Xin X., Zhao B., He Y., Hu Y., Liu S. Design and analysis of a haptic master manipulator for minimally invasive surgery. Proceedings of the 2017 IEEE International Conference on Information and Automation (ICIA).

[16] Takei M., Shiraiwa H., Omata S., Motooka N., Mitamura K., Horie T., Ookubo T., Sawada S. (2004). A new tactile skin sensor for measuring skin hardness in patients with systemic sclerosis and autoimmune Raynaud’s phenomenon. J. Int. Med. Res..

[17] Zhang L., Ju F., Cao Y., Wang Y., Chen B. (2017). A tactile sensor for measuring hardness of soft tissue with applications to minimally invasive surgery. Sens. Actuators A Phys..

[18] Han Y.-M., Oh J.-S., Kim J.-K., Choi S.-B. (2014). Design and experimental evaluation of a tactile display featuring magnetorheological fluids. Smart Mater. Struct..

[19] Oh J.S., Kim J.K., Lee S.R., Choi S.B., Song B.K. (2013). Design of tactile device for medical application using magnetorheological fluid. J. Phys. Conf. Ser..

[20] Kim S., Kim P., Park C.-Y., Choi S.-B. (2016). A new tactile device using magneto-rheological sponge cells for medical applications: Experimental investigation. Sens. Actuators A Phys..

[21] Groenen M.A.M., Archibald A.L., Uenishi H., Tuggle C.K., Takeuchi Y., Rothschild M.F., Rogel-Gaillard C., Park C., Milan D., Megens H.-J. (2012). Analyses of pig genomes provide insight into porcine demography and evolution. Nature.

[22] Nava A., Mazza E., Furrer M., Villiger P., Reinhart W.H. (2008). In vivo mechanical characterization of human liver. Med. Image Anal..

[23] Carter F.J., Frank T.G., Davies P.J., McLean D., Cuschieri A. (2001). Measurements and modelling of the compliance of human and porcine organs. Med. Image Anal..

[24] Cha S.W., Kang S.R., Hwang Y.H., Oh J.S., Choi S.B. (2018). A controllable tactile device for human-like tissue realization using smart magneto-rheological fluids: Fabrication and modeling. Smart Mater. Struct..

[25] Bland D.R. (2016). The Theory of Linear Viscoelasticity.

[26] MRF-132DG. http://www.lordmrstore.com/lord-mr-products/mrf-132dg-magneto-rheological-fluid.

[27] Tsai L.W., Walsh G.C., Stamper R.E. Kinematics of a novel three DOF translational platform. Proceedings of the IEEE International Conference on Robotics and Automation.

[28] Tavakoli M. (2008). Haptics for Teleoperated Surgical Robotic Systems.

[29] Kim P., Kim S., Park Y.D., Choi S.B. (2016). Force modeling for incisions into various tissues with MRF haptic master. Smart Mater. Struct..

[30] Han Y.M., Oh J.S., Kim S., Choi S.B. (2017). Design of multi-degree motion haptic mechanisms using smart fluid-based devices. Mech. Based Des. Struct. Mach..

[31] Kang S.R. (2018). Design and Control of 6-DOF Haptic Master Using MR Fluid for Robot Surgery. Master’s Thesis.

[32] Hwang Y.H. (2018). Design of 7-DOF Slave Robot Integrated with Magneto-Rheological Haptic Master. Master’s Thesis.

